# Supplementary material for: Quorum Quenching Strains Isolated from the Microbiota of Sea Anemones and Holothurians Attenuate Vibrio corallilyticus Virulence Factors and Reduce Mortality in Artemia salina
Source: Microorganisms. 2022 Mar 16;10(3):631. doi: 10.3390/microorganisms10030631 (PMC8950658; doi:10.3390/microorganisms10030631)
Supplement: Supplementary file 1 [file microorganisms-10-00631-s001.zip › microorganisms-1614438-supplementary.pdf]

microorganisms

MDPI

Supplementary Materials

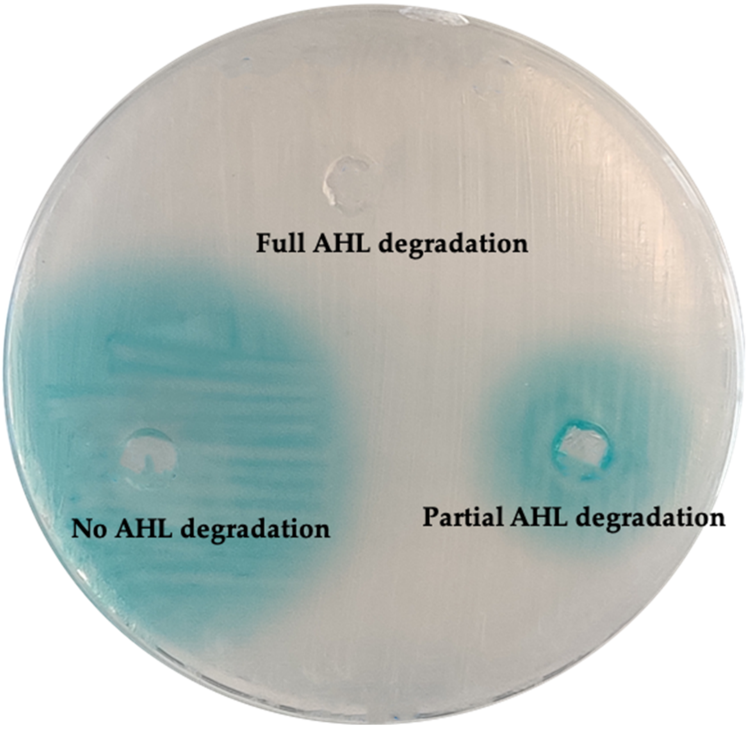

Figure S1. Diffusion agar-plate assay to detect AHLs using the biosensor *Agrobacterium tumefaciens* NTL4 (pZLR4). The size of halo diameter indicate AHL degradation activity.

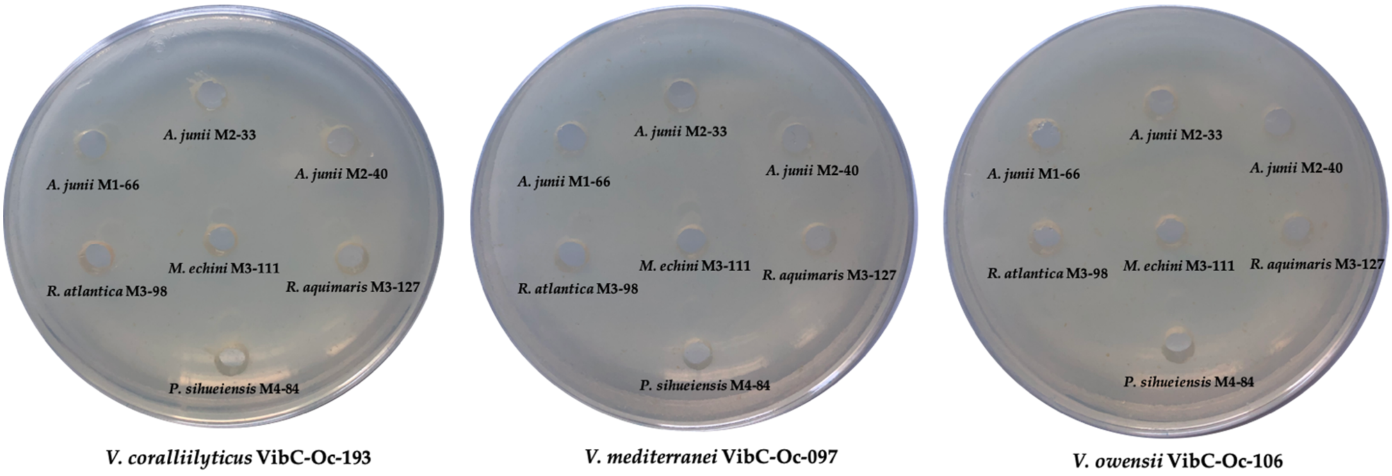

Figure S2. Determination of remaining AHLs after 24 h incubation of cocultures with AHL-degrading strains and *Vibrio* spp. using diffusion agar-plate assay with *Agrobacterium tumefaciens* NTL4 (pZLR4).

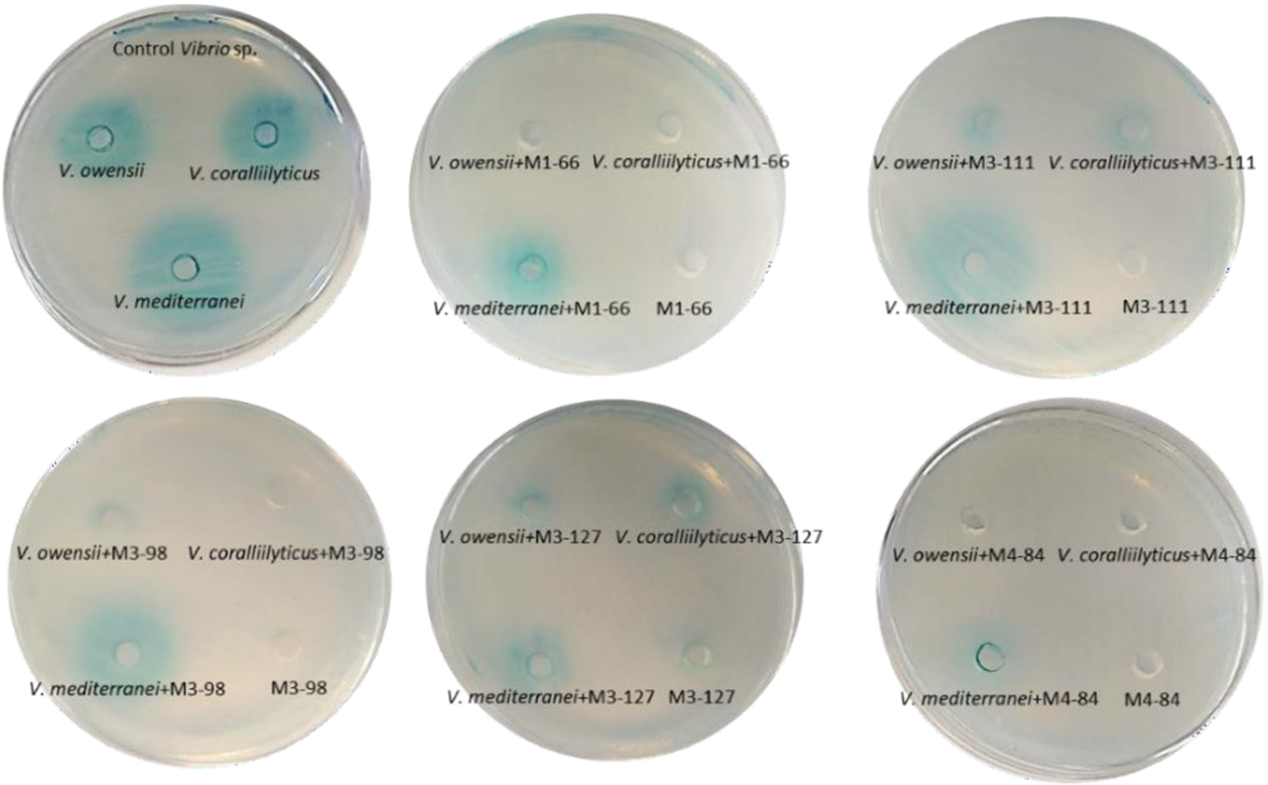

Figure S3. Detection of growth inhibition of *Vibrio* spp. by the AHL-degrading strains. Growth inhibition was detected by loading 100-μL aliquots of cell-free supernatant of each AHL-degrading culture strain in wells made on the surface AM medium plates. An overlay of the strains *V. coralliilyticus* VibC-Oc-193, *V. mediterranei* VibC-Oc-097 and *V. owensii* VibC-Oc-106 was previously placed, respectively.

Table S1. Phenotypes of *V. coralliilyticus* VibC-Oc-193 after co-culturing with AHL-degrading strains.

	Amylase	Hemolysin	swarming	swimming	DNAase	Caseinase	Tween 20	Alkaline phosphatase
**VibC-Oc-193**	++	++	++	+++	++	++	++	++
***Acinetobacter junii* M1-66**	-	-	-	-	-	-	++	+
***A.junii* M1-66 + VibC-Oc-193**	+	-	++	+++	++	++	++	++
***A. junii* M2-33**	+	-	++	++	++	++	++	-
***A.junii* M2-33 + VibC-Oc-193**	+	+	++	+++	++	++	++	++
***A.junii* M2-40**	+	-	-	-	+	-	++	+
***A.junii* M2-40 + VibC-Oc-193**	++	++	++	+++	+	++	++	++
***Ruegeria atlantica* M3-98**	+	-	-	-	-	-	-	+
***R. atlantica* M3-98 + VibC-Oc-193**	++	+	++	+++	++	++	++	++
***Microbulbifer echini* M3-111**	+	++	-	++	++	+	++	++

M. echini **M3-111 + VibC-Oc-193**	+	++	-	++	++	++	++	++
Rheinheimera aquimaris **M3-127**	++	-	++	++	++	++	++	+
R. aquimaris **M3-127 + VibC-Oc-193**	++	+	++	+++	++	++	++	++
Pseudomonas sihuiensis **M4-84**	-	-	++	++	-	-	++	+
P. sihuiensis **M4-84 + VibC-Oc-193**	-	-	++	+++	+	++	++	+

"+++ and ++" strong activity, "+" medium activity and "-" no activity
